# The Superiority of Zoledronic Acid Over Risedronate for Paget’s Disease: A 16-Year Experience at a Single Institution

**DOI:** 10.7759/cureus.32923

**Published:** 2022-12-25

**Authors:** Christiana Zidrou, Stavroula Rizou, Anastasios Beletsiotis

**Affiliations:** 1 2nd Orthopedic Department, General Hospital of Thessaloniki “Papageorgiou”, Thessaloniki, GRC; 2 Medicine Department, National and Kapodistrian University of Athens School of Medicine, Athens, GRC; 3 Orthopedic Department, General Hospital of Thessaloniki "Papageorgiou", Thessaloniki, GRC

**Keywords:** bone, zoledronic acid, treatment, risedronate, paget’s disease

## Abstract

Introduction

Bisphosphonates are considered to be the treatment of choice for patients with active Paget’s disease. The aim of this study was to record and assess the therapeutic effect in response to a single intravenous infusion of 5mg zoledronic acid or oral risedronate.

Methods

A retrospective observational study was conducted of 89 patients in Greek patients with active Paget’s disease from a tertiary hospital in North Greece. Patients were treated with either a single intravenous infusion of 5mg zoledronic acid (1st group, n=46) or 30mg of risedronate per day for 60 days (2nd group, n=43). All patients received 1000mg of calcium and 400-800IU of calciferol daily. The primary outcome measure was to record the therapeutic response defined as the control of patients' symptoms and normalization of the biochemical markers of bone metabolism. The secondary outcome measures included the patient's quality of life which was evaluated by the questionnaire SF-36 and adverse events.

Results

Forty patients from the zoledronic acid group and 38 patients from the risedronate group, who reported pain at the beginning of the study, showed a significant degree of clinical improvement. All the patients in our study showed a therapeutic response at six months while the remission was persistent at 36 months. There was a statistically significant difference between pre-treatment basal results and values at the sixth and 36th months of treatment (p<0.001) between the two groups.

Conclusion

The study demonstrated the superiority of zoledronic acid over risedronate in patients with active Paget’s disease.

## Introduction

Paget’s disease of bone is a chronic metabolic bone disorder, of unknown etiology, with increased bone turnover markers resulting in defective bone microarchitecture. This results in loss of structural integrity, arthritis, deformity of the skull and long bones, hearing loss, and pathological fractures [1]. It is more common in people of European descent [2] and rarely occurs in Asia and Africa [3]. Paget’s disease is usually asymptomatic and is diagnosed randomly in people undergoing radiological examination for another cause [4]. Serum alkaline phosphatase (ALP) is a sensitive indicator of the activity and the severity of the disease, that can be used for the diagnostic screening of patients. Bone scintigraphy is particularly valuable in specifying the extent of the disease. Both parameters are particularly useful for monitoring response to treatment and controlling recurrence.

In active Paget’s disease, the main goal of treatment is to suppress accelerated bone metabolism. Bisphosphonates have been used for this purpose for decades [5]. Bisphosphonates reduce disease activity and severity, improve symptoms, and normalize bone metabolism markers [6]. Many studies have demonstrated that intravenous zoledronic acid is superior to other oral bisphosphonates [7,8]. The response to zoledronic acid is more rapid, complete, and sustained compared to oral bisphosphonates [9]. Recent guidelines suggest that treatment with a single infusion of zoledronic acid 5mg is the treatment of choice for active Paget’s disease [10].

Paget’s disease is rare in Greece. There is a lack of long-term follow-up data on the superiority of zoledronic acid response compared to oral risedronate in this population. Thus, the primary goal of our study was to record and evaluate the therapeutic effect of 5mg intravenous zoledronic acid and oral risedronate in Greek patients with active Paget’s disease.

## Materials and methods

Study design

This was a single-center, retrospective, observational study carried out at a tertiary Greek care hospital in Thessaloniki, over a 16-year period (from December 2005 to November 2021).

Study population

All patients, who were older than 55 years old, had active Paget’s disease of bone (PDB), and attended the metabolic bone disease clinic of G. Papageorgiou General Hospital, Orthopedic Department were included in the study. The diagnosis of Paget’s disease was confirmed by clinical evaluation, patient history, blood tests (ALP), and x-rays. The exclusion criteria were as follows: (i) serum 25-hydroxyvitamin D level <15nmol/l, (ii) hepatic or renal disease, (iii) primary hyperparathyroidism, (iv) a history of iritis, uveitis, or retinopathy, (v) diabetic nephropathy, (vi) a history of upper gastrointestinal disorders that may affect the compliance with the protocol, (vii) the use of therapy for Paget’s disease the previous six months.

During the study period, 91 patients had confirmed active PDB. Two patients were excluded from the study since they were lost to follow-up before six months of treatment. The study population was 46 patients who received a single intravenous infusion of 5mg zoledronic acid (1st group) and 43 patients who received 30mg of risedronate per day for 60 days (2nd group). The difference in treatment was due to the different time study periods in that patients had PDB. All patients received 1000mg of calcium and 400-800IU of calciferol per day.

Data collection and outcome measures

The main outcome measure was to record the therapeutic responses in both groups. Normalization of ALP levels or a reduction of at least 75% in ALP excess (the difference from the midpoint of the reference range, normal range: 40-129U/l) at six months was defined as a therapeutic response. Return of serum ALP levels to within 20% of the pre-treatment basal levels after the therapeutic response was considered a relapse [9]. Pre-treatment, six- and 36-months post-treatment serum ALP levels were available for all 89 patients.

The secondary outcome measures included patients' biochemical markers of bone resorption (by the serum levels of b-C telopeptide of type I collagen (bCTx)) and bone formation (by the serum levels of N-terminal propeptide of type I collagen (P1NP)) [11], the quality of life, and adverse events. The quality of life was measured by the use of SF-36 (Short-Form General Health Survey) which evaluates eight aspects of health status: physical and social functioning, physical and emotional roles, general and mental health, bodily pain, and vitality. Scores on each SF-36 scale can range from 0 (worst) to 100 (best), with higher scores indicating a better quality of life [12].

Clinical improvement was evaluated based on follow-up records showing the estimation of pain reduction based on patient self-assessment compared to the severity of the onset pain. The radiological skeletal examination was conducted in all patients pre-treatment, at six- and 36-months of follow-up and X-rays were evaluated by an experienced radiologist. Bone scintigraphy was performed in all patients to determine the extent of the disease pre-treatment, at six and 36 months of follow-up after administration of zoledronic acid or risedronate. Eleven transiliac bone biopsies were performed while the patient was under local anesthesia, having obtained two doses of tetracycline. Biopsy specimens had to contain both cortexes with intact trabecular bone, in order to be suitable for quantitative histomorphometry.

Ethics approval

The study was performed in line with the World Medical Association Declaration of Helsinki-Ethical Principles for Medical Research Involving Human Subjects and the ethical approval for the study was obtained from the Scientific Committee of G. Papageorgiou Hospital in Greece (372/May 2022).

Statistical analysis

The data were analyzed by using Statistical Product and Service Solutions (SPSS) (IBM SPSS Statistics for Windows, Version 26.0, Armonk, NY). Continuous variables (age, weight, serum ALP, serum levels of bCTx, serum levels of P1NP, aspects of SF-36 score) are expressed as mean± standard deviation (SD) while categorical variables (male gender, previous therapy for Paget’s disease, adverse events, site of Paget involvement) are expressed as counts and percentages. The analysis was repeated for the subgroups based on age, gender, weight, previous therapy for Paget’s disease, aspects of SF-36 score, rate of adverse events, and site of Paget involvement. Pearson’s Chi-square test was used for the evaluation of the association between zoledronic acid or risedronate administration and categorical variables. Mann-Whitney test was performed for unifactorial analysis so as to evaluate the association between zoledronic acid or risedronate administration and continuous variables in case of violation of normality. Statistical significance was set at p<0.05.

## Results

Baseline parameters

Data from 89 patients with active Paget disease were analyzed retrospectively. The mean age of the patients was 73.9±6.0 years in the zoledronic acid group and 73.5±4.1 years in the risedronate group (Table 1). There were 32 males and 14 females in the zoledronic acid group while there were 29 males and 14 females in the risedronate group. Twenty-four patients from the zoledronic acid group and 22 from the risedronate group were recently diagnosed and received no therapy before the initiation of the study. The most frequently reported symptom (88%) was body pain. Four patients from the zoledronic acid group and three from the risedronate group reported a particularly high ALP value as a random finding, with normal indicators of liver function.

**Table 1:** Baseline characteristics of the patients. P1NP: N-terminal propeptide of type I collagen; bCTx: b-C telopeptide of type I collagen; SF-36: Short Form Health Survey

Characteristics	Zoledronic acid n=46	Risedronate n=43	P-value
Age (years)	73.9±6	73.5±4.1	0.799
Gender			0.655
Male (no,%)	32 (69.5)	29 (67.4)	
Female (no,%)	14 (30.5)	14 (32.6)	
Weight (kg)	82.4±6	82.7±6.6	0.653
Previous therapy for Paget’s disease (no,%)			0.991
Risedronate	5 (10.9)	4 (9.3)	
Alendronate	4 (8.7)	5 (11.6)	
Other oral bisphosphonates	6 (13.1)	6 (14)	
Intravenous bisphosphonates	7 (15.2)	6 (14)	
None	24 (52.1)	22 (51.1)	
Serum alkaline phosphatase (40-129U/l) Basal	395.1±139.4	371.4±139.7	0.405
				Serum P1NP (μg/l) Basal	454.1±50.8	425.2±28.9	0.004
								Serum bCTx (nmol/l) Basal	13±1.5	12.6±1.7	0.220
SF-36 score											
Physical functioning	57±4	61±3	0.028
Physical role	58±4.8	63±4.3	0.031
Bodily pain	59±3.1	59±5.3	0.250
General health	63±4.4	65±3.7	0.361
Vitality	55±4	56±2.9	0.198
Social functioning	77±2.5	79±2.6	0.393
Emotional role	72±2.9	76±0.34	0.002
mental health	76±2.9	77±1.8	0.621
Physical component summary	39±3	41±3	0.483
Mental component summary	52±2.7	52±3	0.705

Bone deformities were recorded in 14 patients (30,4%) from the zoledronic acid group and in 13 ones (30,2%) from the risedronate group. Especially, seven patients from the zoledronic acid group and seven from the risedronate group had bowing of the tibia, four patients from the zoledronic acid group and three from the risedronate group had a deformity of the shaft of the femur, while three patients from the zoledronic acid group and two from the risedronate group had frontal bossing. Thirteen patients (seven from the zoledronic acid group and six from the risedronate group) had fissure fractures at the beginning of the study. Forty-four patients (23 from the zoledronic acid group and 21 from the risedronate group) had audiometrically confirmed hearing loss. Osteoporosis was diagnosed in 34 patients (18 from the zoledronic acid group and 16 from the risedronate group) while dyslipidemia was recorded in 43 patients (23 from the zoledronic acid group and 20 from the risedronate group). Forty-five patients (24 from the zoledronic acid group and 21 from the risedronate group) suffered from hypertension. Basal serum ALP level was 395.1±139.4U/L and 371.4±139.7U/L in the zoledronic acid and risedronate group retrospectively.

Clinical and biochemical response

Patients who reported pain at the beginning of the study (N=40 from the zoledronic acid group and N=38 from the risedronate group), showed significant clinical improvement. In the zoledronic acid group, mean scores for each of the eight components of the SF-36 had an upward trend at both six and 36 months, indicating the quality of life improvements, whereas in the risedronate group the responses were varied (Table 2). There was a significantly greater improvement in the zoledronic acid group compared to the risedronate group in terms of physical functioning at six months and general health and bodily pain at six and 36 months. Furthermore, the zoledronic acid group had a significant improvement from baseline scores in the physical and mental component summary scores at both six and 24 months.

**Table 2:** Quality of life according to the SF-36 score at six and 36 months. SF-36: Short Form Health Survey

SF-36 score	Zoledronic acid			Risedronate				
	Basal	6 months	36 months	Basal	6 months	36 months	P-value at 6 months	P-value at 36 months
Physical functioning	57±4	65±3.8	63±3.7	61±3	63±3.1	61±3.1	0.001	0.025
Physical role	58±4.8	63±4.4	63±4.4	63±4.3	63±4.4	63±4.5	0.321	0.821
Bodily pain	59±3.1	71±3.2	70±3.1	59±5.3	64±5.1	61±4.8	<0.001	<0.001
General health	63±4.4	73±3.4	71±3.5	65±3.7	67±3	66±3.2	<0.001	<0.001
Vitality	55±4	67±3.1	65±2.8	56±2.9	60±2.76	57±2.8	0.103	0.088
Social functioning	77±2.5	88±2.5	85±2.8	79±2.6	82±2.5	80±2.4	0.963	0.927
Emotional role	72±2.9	80±3.1	79±3.2	76±0.34	78±2.8	77±2.9	0.085	0.089
Mental health	76±2.9	83±2.3	82±2.4	77±1.8	79±2.2	78±1.2	<0.001	<0.001
Physical component summary	39±3	50±3	48±2.8	41±3	43±2.9	42±3	<0.001	<0.001
Mental component summary	52±2.7	63±3.3	61±3.2	52±3	54±2.8	53±2.9	<0.001	<0.001

Pre-treatment results of bone turnover markers (serum ALP, serum P1NP, and serum bCTx) at six and 36 months are shown in (Table 3). All the patients in our study showed a therapeutic response at six months while the remission was persistent at 36 months. There was a statistically significant difference between pre-treatment basal results and values at the sixth and 36th months of treatment (p<0.001).

**Table 3:** Bone turnover markers concentration at six and 36 months. Serum ALP, P1NP, and bCTx values at six and 36 months were significantly lower than basal values. ALP: alkaline phosphatase; P1NP: N-terminal propeptide of type I collagen; bCTx: b-C telopeptide of type I collagen

	Zoledronic acid			Risedronate				
	Basal	6 months	36 months	Basal	6 months	36 months	P-value at 6 months	P-value at 36 months
Serum ALP (U/L)	395.1±139.4	78,4±10.3	84±9	371.4±139.7	92.7±13.6	143.4±19.1	<0.001	<0.001
Serum P1NP (μg/l)	454.1±50.8	37.8±7.1	39.5±6.5	425.2±28.9	64±6.7	117.4±15.2	<0.001	<0.001
Serum bCTx (nmol/l)	13±1.5	2.8±0.5	2.9±0.4	12.6±1.7	5.9±0.7	7.8±0.8	<0.001	<0.001

Scintigraphic response

Before treatment, bone scintigraphy identified 178 lesions in 46 patients (zoledronic acid group) and 162 lesions in 43 patients (risedronate group) (Table 4). Polyostotic was present in all patients, and the pelvis was the most frequent location of the disease. Skull involvement was observed in 36 patients (19 from the zoledronic acid group and 17 from the risedronate group) and all of them had increased levels of basal serum ALP.

**Table 4:** Site of Paget involvement.

	Zoledronic acid group n=46	Risedronate group n=43
Site	No of patients, %	No of patients, %
Pelvis	42/46, (91.3)	39/43, (90.7)
Spine	33/46, (71.7)	30/43(69.8)
Femur	29/46, (63.0)	25/43, (58.1)
Tibia	16/46, (34.8)	14/43, (32.6)
Skull	19/46, (42.3)	17/43, (39.5)
Humerus	12/46, (26.0)	10/43, (23.2)
Scapula	8/46, (17.4)	7/43, (16.3)
Clavicle	4/46, (8.7)	4/43, (9.3)
Ribs	7/46, (15.2)	8/43, (18.6)
Maxilla	4/46, (8.7)	4/43, (9.3)
Mandible	4/46, (8.7)	4/43, (9.3)
Spine	33/46	30/43
Cervical	3/33, (9.0)	2/30, (6.7)
Thoracic	12/33, (36.4)	10/30, (33.3)
Lumbar	13/33, (39.4)	11/30, (36.7)
Sacrum	5/33, (15.2)	7/30, (23.3)

Follow-up bone scintigraphy at six and 36 months was conducted in 40 patients in the zoledronic acid group and in 38 patients in the risedronate group. Six patients from the zoledronic acid group and five patients from the risedronate group refused to repeat the bone scan. A follow-up bone scan at 36 months showed that 16 lesions from the zoledronic acid group and 13 lesions from the risedronate group were healed completely, while the remaining lesions showed a significant degree of scintigraphic improvement.

Bone biopsy

Transiliac bone biopsies were conducted in 11 patients, specifically six in the zoledronic acid group and five in the risedronate group. Four biopsy samples were insufficient but showed no abnormalities in the quality examination. Of the seven biopsy samples that could be assessed, two came from sites of Paget’s involvement (one sample from each group) and were normal apart from the enhanced mineralizing surface in the patient from the risedronate group. The remaining five samples from non-Pagetic bone were similar, with the only difference being that the extent of the mineralizing surface was decreased in the zoledronic acid group.

Adverse events

No deaths were documented. The number of patients with adverse events (44 in the zoledronic acid group and 33 in the risedronate group) was recorded. Because there was a marked increase in side effects in the first three days after intravenous administration of the zoledronic acid, the data were tabulated separately for this period (Table 5). In the first three days, the zoledronic acid group had twice the number of side effects compared to the risedronate group (p=0.009). These were mainly the symptoms of flu-like illness, following zoledronic acid intravenous administration. The majority of symptoms declined within three to four days by taking oral paracetamol. Then the rates of adverse events were similar in both groups. Atrial fibrillation, renal impairment, and hypophosphatemia were not reported. Hypocalcemia was developed in five patients in the zoledronic acid group and was asymptomatic in four of these. The one patient with mild symptoms had not taken his calcium and vitamin D supplements.

**Table 5:** Timing and rates of adverse events.

Adverse events	Zoledronic acid n=46	Risedronate n=43	P-value
Days 1-3, n %	25 (54.3)	12 (27.9)	0.009
Influenza-like illness	5 (10.9)	2 (4.6)	0.263
Pyrexia	4 (8.7)	1 (2.3)	0.184
Myalgia	4 (8.7)	2 (4.6)	0.430
Fatigue	3 (6.5)	1 (2.3)	0.328
Headache	3 (6.5)	2 (4.6)	0.683
Rigors	3 (6.5)	2 (4.6)	0.683
Nausea	3 (6.5)	1 (2.3)	0.328
Bone pain	2 (4.3)	1 (2.3)	0.584
After study day 3, n %	19 (41.3)	21 (48.8)	0.056
Pain in arm or leg	4 (8.7)	3 (6.9)	<0.001
Arthralgia	3 (6.5)	5 (11.6)	0.672
Dizziness	3 (6.5)	1 (2.3)	0.328
Nasopharyngitis	3 (6.5)	4 (9.3)	0.648
Diarrhea	2 (4.3)	2 (4.6)	0.683
Headache	2 (4.3)	3 (6.9)	0.608
Backpain	2 (4.3)	3 (6.9)	0.963

## Discussion

Our study clarifies the pattern of presentation of patients with active Paget disease in the Greek population, especially from North Greece. The study suggested the superiority of intravenous 5mg zoledronic acid over oral risedronate in therapeutic response in patients with active Paget’s disease and this response was sustained for three years. This is in line with findings from previous studies.

Reid et al. suggested that a single infusion of zoledronic acid is superior to oral risedronate in patients with active Paget’s disease [7]. It is more effective and demonstrates more rapid and sustained long-term remission compared to risedronate in patients with active Paget’s disease [7]. Baykan et al. in a retrospective study of 12 patients suggested that the remission of the disease occurred at six months and persisted for up to 18 months after a single intravenous infusion of zoledronic acid [13]. Moreover, in another study, Tucci et al. recorded the effect of zoledronic acid in 14 patients with active Paget’s disease who had previously received other bisphosphonates but no remission of the disease had been achieved or not maintained for more than 12 months [14].

All patients achieved a significant improvement of clinical symptoms and a reduction in biochemical markers, to a greater extent in the zoledronic acid group compared to the risedronate group, after six months of treatment. Moreover, all patients who had completed three years of follow-up were still in remission to a greater extent in the zoledronic acid group compared to the risedronate group. The prolonged normalization of bone turnover markers that is achieved to a greater extent in the zoledronic acid group compared to the risedronate group has been proven to be responsible for reducing symptoms and complications [15].

Serum ALP is an indicator of the overall assessment of disease activity and severity. It does not provide information on the activity of individual lesions that can be scintigraphically evaluated. Avramidis et al. evaluated scintigraphic data quantitatively in nine patients and suggested that the scintigraphic response was achieved as early as the first three months and was maintained for at least 12 months after a single intravenous infusion of zoledronic acid [16]. As mentioned above, in our study, a follow-up bone scan at 36 months showed that 16 lesions from the zoledronic acid group and 13 lesions from the risedronate group were healed completely, while the remaining lesions showed a significant degree of scintigraphic improvement.

Intravenous bisphosphonates cause an acute phase characterized by influenza-like syndrome after the first dose in 10-30% of patients. The main causal factor of the phenomenon is believed to be the production of pro-inflammatory cytokines [17]. In our study, five patients from the zoledronic acid group and two patients from the risedronate group suffered from a flu-like syndrome which was improved by oral paracetamol administration.

The limitations of our study are the limited duration of follow-up (only three years), its origin from one center in North Greece, and its retrospective design. Despite these, we documented the superiority of a single therapeutic dose of zoledronic acid over risedronate in Greek patients with active Paget’s disease in North Greece.

## Conclusions

To the best of our knowledge, this study is one of the very few studies in Greece that documents the superiority of zoledronic acid over risedronate in patients with active Paget disease. The strengths of our study are the availability of three bone turnover markers (serum ALP, serum P1NP, and serum bCTx), relatively large sample size and in addition to clinical and biochemical evaluation, scintigraphic findings were evaluated.
